# Integrated Transcriptome and Metabolome Analysis Reveals the Resistance Mechanisms of *Brassica napus* Against *Xanthomonas campestris*

**DOI:** 10.3390/ijms26010367

**Published:** 2025-01-03

**Authors:** Cong Zhou, Li Xu, Rong Zuo, Zetao Bai, Tongyu Fu, Lingyi Zeng, Li Qin, Xiong Zhang, Cuicui Shen, Fan Liu, Feng Gao, Meili Xie, Chaobo Tong, Li Ren, Junyan Huang, Lijiang Liu, Shengyi Liu

**Affiliations:** Key Laboratory of Biology and Genetics Improvement of Oil Crops, Oil Crops Research Institute of Chinese Academy of Agricultural Sciences, Ministry of Agriculture and Rural Affairs, Wuhan 430062, China; zhoucong@caas.cn (C.Z.);

**Keywords:** rapeseed, *Xcc*, transcriptome, metabolome, indole glucosinolates, IAA

## Abstract

Rapeseed (*Brassica napus* L.) is an important crop for healthy edible oil and stockfeed worldwide. However, its growth and yield are severely hampered by black rot, a destructive disease caused by *Xanthomonas campestris* pv. *campestris* (*Xcc*). Despite the identification of several quantitative trait loci (QTLs) associated with resistance to black rot in *Brassica* crops, the underlying molecular mechanisms remain largely unexplored. In this study, we investigated *Xcc*-induced transcriptomic and metabolic changes in the leaves of two rapeseed varieties: Westar (susceptible) and ZS5 (resistant). Our findings indicated that *Xcc* infection elicited more pronounced overall transcriptomic and metabolic changes in Westar compared to ZS5. Transcriptomic analyses revealed that the phenylpropanoid biosynthesis, cutin, suberine and wax biosynthesis, tryptophan metabolism, and phenylalanine metabolism were enriched in both varieties. Notably, photosynthesis was down-regulated in Westar after infection, whereas this down-regulation occurred at a later stage in ZS5. Integrated analyses of transcriptome and metabolome revealed that the tryptophan metabolism pathway was enriched in both varieties. Indolic glucosinolates and indole-3-acetic acid (IAA) are two metabolites derived from tryptophan. The expression of genes involved in the indolic glucosinolate pathway and the levels of indolic glucosinolates were significantly elevated in both varieties post-infection. Additionally, exogenous application of IAA promoted the development of black rot, whereas the use of an IAA synthesis inhibitor attenuated black rot development in both resistant and susceptible rapeseed varieties. These findings provide valuable molecular insights into the interactions between rapeseed and *Xcc*, facilitating the advancement of black rot resistance breeding in *Brassica* crops.

## 1. Introduction

Rapeseed (*Brassica napus* L.) was originally formed approximately 7500 years ago by natural hybridization between the ancestors of *Brassica rapa* and *Brassica oleracea* [1]. Rapeseed contributes roughly 13% to 16% of the global vegetable oil supply and holds significant economic value as an oilseed crop worldwide [2]. Black rot, caused by the bacterial pathogen *Xanthomonas campestris* pv. *campestris* (*Xcc*), is one of the most serious and destructive diseases in *Brassica* crops such as *B. napus*, *B. rapa*, and *B. oleracea* [3]. Typical disease symptoms of *Xcc* infection include necrotic, darkened leaf veins and V-shaped yellow lesions starting from the leaf margins, resulting in substantial yield losses and reduced quality in *Brassica* crops [4,5]. Although various physical methods, chemical treatments, and biological control strategies can inhibit the activity or pathogenicity of *Xcc*, breeding and deployment of black-rot-resistant varieties remains the most effective and sustainable strategy for disease management [6]. A comprehensive understanding of plant defense mechanisms against biotic stresses is crucial for the development of resistant crop cultivars [7]. Nevertheless, the molecular mechanisms underlying resistance to black rot in *Brassica* crops remain largely unexplored.

In recent decades, more than 60 QTLs associated with resistance to black rot have been identified in *B. oleracea*, *B. rapa*, *B. carinata*, and *B. napus* [8,9]. However, the genes conferring resistance to black rot have not been cloned in *Brassica* crops. Previous studies have indicated correlations between various secondary metabolites such as glucosinolates, flavonoids, hydroxycinnamic acids, and phenolics, and the infection by *Xcc* in *Brassica* species [10,11,12]. Several studies employing omics approaches have revealed the differential responses of genes and metabolites in susceptible and resistant *Brassica* cultivars in response to black rot [8]. Transcriptome analysis of two *B. rapa* cultivars with contrasting resistance levels identified the top ten differentially expressed genes (DEGs), which include NBS-LRR-type genes, protein kinase genes, and expansin genes, indicating their potential role in the response to black rot [13]. During *Xcc* attack on *B. oleracea* plants, up-regulation of genes involved in terpenes, flavonoids, alkaloids, anthocyanins, salicylic acid (SA), ethylene, and jasmonic acid (JA) pathways was observed, indicating their importance during pathogenesis [14]. Similarly, transcriptomic analyses of two *B. oleracea* lines highlighted the importance of genes involved in the glucosinolate pathway, catabolic pathways, reactive oxygen species (ROS) scavenging, and photosynthetic metabolism for *Xcc* resistance [15]. Furthermore, alkaloids, coumarins, and sphingolipids played crucial roles in the *Xcc* infection process in *B. oleracea* [16]. An integrated metabolome and transcriptome analysis revealed a systemic reprogramming of pathogen perception, hormone metabolism, sugar metabolism, and phenylpropanoid metabolisms in *B. oleracea* following *Xcc* infection [17]. Despite the considerable advancements in elucidating the molecular mechanisms underlying black rot resistance in *Brassica* crops, limited research focused on the mechanisms of resistance to *Xcc* in rapeseed.

In plants, tryptophan (Trp) serves as a precursor for a wide range of secondary metabolites, including indole-3-acetic acid (IAA), indole glucosinolates, and camalexin [18,19]. Initially, Trp can be transformed into indole-3-pyruvic acid (IPyA), indole-3-acetamide (IAM), and indole-3-acetaldoxime (IAOx). These intermediates can subsequently undergo enzymatic conversion to form IAA individually. IAA is the predominant form of auxin in most plants, including rapeseed. In rice plants, overexpressing *GH3-8* resulted enhanced resistance against *Xoo* (*Xanthomonas oryzae* pv *oryzae*) by preventing free IAA accumulation and inhibiting expansin expression [20]. An increase in IAA levels can promote disease development during plant–pathogen interactions [21]. IAOx is a well-known precursor of both indole glucosinolates and camalexin, which function as defense metabolites in plants [19]. Two major types of glucosynolates—aliphatic and indolic—are predominantly present in *Brassica* species [22]. The defensive role of indole glucosinolates and their breakdown products is indicated by their increased accumulation in response to pathogens and herbivores in *Brassicaceae* [23]. For instance, the accumulation of indole glucosinolates in infected rapeseed may restrict the spread of *Alternaria brassicae*, particularly in younger leaves [24]. However, the role played by tryptophan metabolism pathway along with its metabolites such as indole glucosinolates and IAA in black rot resistance remains largely unknown.

In this research, we conducted a comprehensive analysis of the transcriptome and metabolome to investigate the alterations caused by *Xcc* infection in both *Xcc*-susceptible and *Xcc*-resistant varieties of rapeseed. By identifying critical genes, metabolites, and metabolic pathways, we systematically elucidated the molecular mechanisms underlying resistance of rapeseed to *Xcc*. Furthermore, we validated the involvement of IAA in the response of rapeseed to *Xcc* infection. Our findings enhance the understanding of tryptophan metabolism in rapeseed plants during *Xcc* infection and provide valuable molecular insights for breeding *Xcc*-resistant varieties.

## 2. Results

### 2.1. Phenotypes of the Resistant and Susceptible Rapeseed Varieties After Xcc Infection

**Figure 1:**
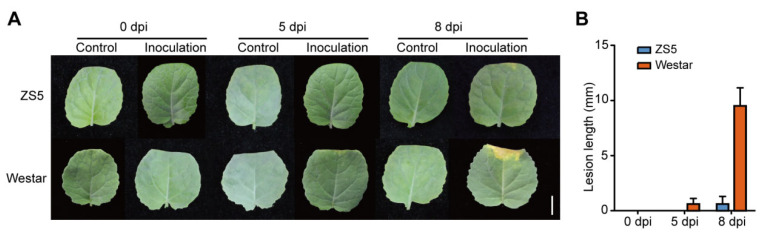
Black rot symptoms on the *B. napus* varieties ZS5 and Westar. (**A**) Phenotypes for ZS5 and Westar leaves at 0, 5, and 8 days (left to right) post-inoculation with *Xcc*. (**B**) Histograms of lesion length for ZS5 and Westar. The control group was inoculated with ddH_2_O. Scale bar = 1 cm.

The phenotypic differences between the resistant (R) rapeseed variety ZS5 and the susceptible (S) rapeseed variety Westar in response to *Xcc* infection are shown in Figure 1. In this study, three leaves per plant at the four-leaf stage were inoculated by clipping leaf tips with scissors that had been dipped in bacterial suspension. At 5 days post-inoculation (dpi), disease symptoms occurred around the leaf edge in Westar, while no visual disease symptoms were detected in ZS5. By 8 dpi, severe V-shaped chlorosis (approximately 10 mm in length) was observed on leaves of Westar, while only minor disease symptoms occurred at the inoculation site in ZS5 (Figure 1). These findings revealed that ZS5 exhibited a higher level of resistance to *Xcc* compared to Westar.

### 2.2. Transcriptional Analysis of Rapeseed in Response to Xcc Infection

**Figure 2:**
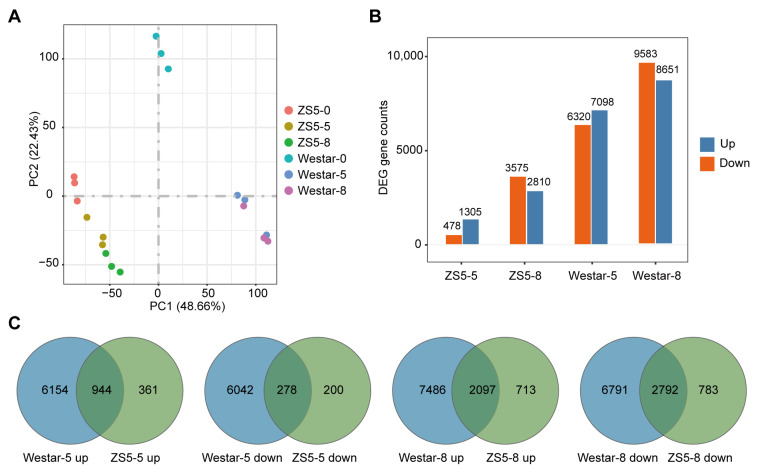
Overview of transcriptomic analysis of Westar and ZS5 in response to *Xcc* infection. (**A**) Principal component analysis (PCA) based on gene expression profiles for the six groups of samples. (**B**) The numbers of DEGs that were up-regulated and down-regulated in Westar and ZS5 at 5 dpi and 8 dpi. (**C**) Venn diagrams illustrate the number of up-regulated and down-regulated DEGs in the two varieties at 5 dpi and 8 dpi.

To investigate the molecular mechanisms underlying rapeseed’s defense against *Xcc*, we performed transcriptomic analysis on the leaves of Westar and ZS5 at three time points: 0, 5, and 8 dpi. These samples are referred to as Westar-0, Westar-5, and Westar-8, and ZS5-0, ZS5-5, and ZS5-8, respectively. A total of 112.7 Gb clean data were generated, with the number of clean reads per sample ranging from 38,702,212 to 49,810,876, and a Q30 value exceeding 93.48% (Table S1). Principal component analysis (PCA) based on gene FPKM (fragments per kilobase of transcript per million mapped reads) clearly distinguished the samples from Westar and ZS5, with each group consisting exclusively of three biological replicates specific to that group (Figure 2A). Additionally, correlation analysis of FPKM values demonstrated strong correlations among the three replicates within each group (Figure S1). These results indicated high reproducibility of the RNA-seq data allowing further in-depth analyses.

To characterize transcriptional changes in the two varieties induced by *Xcc* inoculation, we conducted an analysis of differentially expressed genes (DEGs). The identification of DEGs were conducted using adjust *p*-value ≤ 0.05 and |log_2_(Fold Change)| ≥ 1 as thresholds to determine the significance of gene expression differences between samples. This resulted in the comparison of four data pairs: Westar-0 vs. Westar-5, Westar-0 vs. Westar-8, ZS5-0 vs. ZS5-5, ZS5-0 vs. ZS5-8. At 5 dpi, a total of 13,418 DEGs were identified in Westar, including 7098 up-regulated genes and 6320 down-regulated genes. In contrast, only 1783 DEGs were identified in ZS5 with 1305 up-regulated genes and 478 down-regulated genes (Figure 2B and Tables S2 and S3). At 8 dpi, a total of 18,234 DEGs were identified in Westar, including 8651 up-regulated genes and 9583 down-regulated genes (Table S4). Conversely, there were only 6385 DEGs identified in ZS5, including 2810 up-regulated and 3575 down-regulated genes (Figure 2B and Table S5). The number of DEGs at 5 dpi in Westar was more than seven times higher than that observed in ZS5 and nearly three times higher at 8 dpi (Figure 2B). Both Westar and ZS5 showed an increase in the number of up-regulated and down-regulated DEGs from 5 dpi to 8 dpi (Figure 2B).

**Figure 3:**
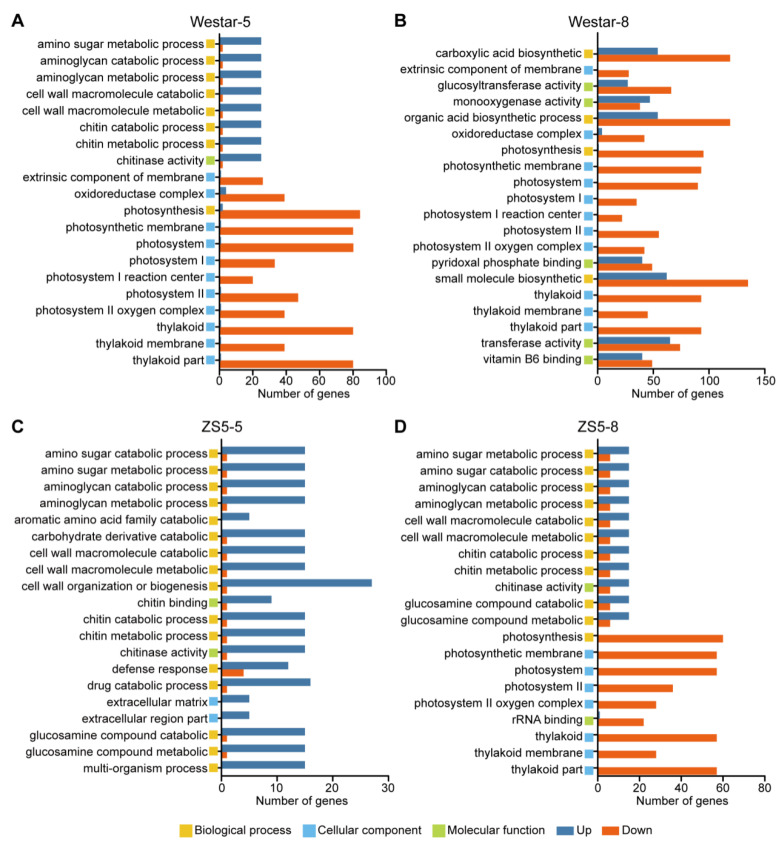
GO (Gene Ontology) enrichment analysis of DEGs detected from the comparisons Westar-0 vs. Westar-5 (**A**), Westar-0 vs. Westar-8 (**B**), ZS5-0 vs. ZS5-5 (**C**), and ZS5-0 vs. ZS5-8 (**D**). The top 20 significant GO terms are presented.

To elucidate the molecular mechanisms of the DEGs, we performed Gene Ontology (GO) analyses on the identified DEGs. The results of the GO enrichment analysis revealed that the DEGs in Westar at 5 dpi were primarily enriched in amino sugar metabolic process, cell wall macromolecule metabolic process, chitinase activity, oxidoreductase complex, and photosynthesis (Figure 3A). At 8 dpi, the DEGs in Westar were primarily enriched in the carboxylic acid biosynthetic process, glucosyltransferase activity, monooxygenase activity, organic acid biosynthetic process, oxidoreductase complex, and photosynthesis. Notably, photosynthesis was down-regulated during both the early and late stages of *Xcc* infection in Westar (Figure 3A,B). In ZS5 at 5 dpi, the DEGs were mainly enriched in amino sugar metabolic process, cell wall macromolecule metabolic process, cell wall organization or biogenesis, chitinase activity, and defense response. Furthermore, nearly all DEGs within the top 20 significant GO terms identified in ZS5 at 5 dpi were up-regulated (Figure 3C). Interestingly, the enriched GO terms in ZS5 at 8 dpi were almost identical to those in Westar at 5 dpi (Figure 3A,D).

Kyoto Encyclopedia of Genes and Genomes (KEGG) analysis revealed that these DEGs were enriched in different pathways between the two varieties (Figure S2). In the ZS5 variety, the DEGs were mainly enriched in phenylpropanoid biosynthesis, cutin, suberine and wax biosynthesis, tryptophan metabolism, phenylalanine metabolism, glucosinolate biosynthesis, and flavonoid biosynthesis at 5 dpi. Notably, nearly all genes within these pathways were up-regulated (Table S6). At 8 dpi in ZS5, most genes were up-regulated in phenylpropanoid biosynthesis and glucosinolate biosynthesis. In contrast, a down-regulation of genes related to photosynthesis was observed (Table S6). Specifically, photosynthesis-related genes were down-regulated in Westar at both 5 dpi and 8 dpi. Moreover, all the pathways enriched in ZS5 overlapped with those identified in Westar except the flavonoid biosynthesis (Figure S2A,B and Table S6). The specific enrichment of the flavonoid biosynthesis pathways in ZS5 indicate their potential role in conferring resistance against *Xcc*.

To identify potential genes associated with resistance to *Xcc*, Venn diagrams were employed to analyze the up- and down-regulated DEGs in the two varieties. In Westar, a total of 6145 DEGs were specifically up-regulated and 6042 DEGs were down-regulated at 5 dpi. Conversely, in ZS5, there were 361 and 200 DEGs that were specifically up- and down-regulated, respectively (Figure 2C). At 8 dpi, Westar exhibited 7486 and 6791 DEGs that were specifically up- and down-regulated, whereas ZS5 showed 713 and 783 DEGs that were specifically up- and down-regulated (Figure 2C). These findings indicate that the susceptible variety Westar and the resistant variety ZS5 exhibit distinct gene expression patterns in response to *Xcc* infection. The unique DEGs might play a critical role in determining the susceptibility or resistance of these varieties to *Xcc*.

### 2.3. Metabolomic Analysis of Rapeseed in Response to Xcc Infection

**Figure 4:**
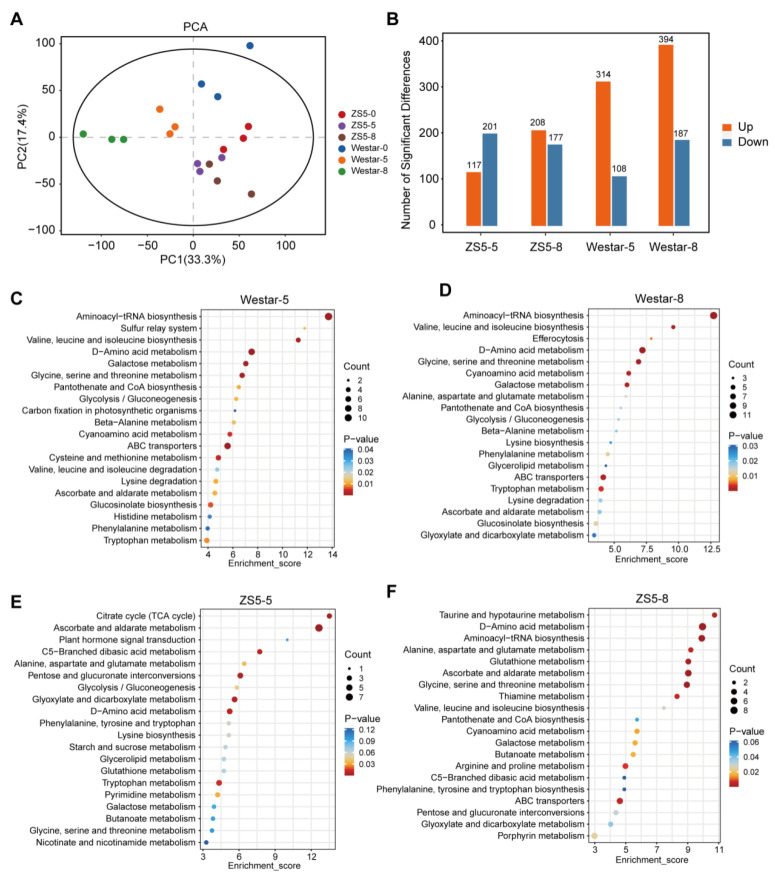
Differentially accumulated metabolites (DAMs) identified from the two rapeseed varieties after *Xcc* infection. (**A**) Principal component analysis (PCA) plots of total detected metabolites. (**B**) The numbers of up- and down-regulated DAMs in the four comparison groups. (**C**–**F**) KEGG pathway analysis of the DAMs in Westar (**C**,**D**) and ZS5 (**E**,**F**) after *Xcc* infection for 5 and 8 days.

To investigate the metabolic responses of the resistant and susceptible rapeseed varieties to *Xcc* infection, metabolites were extracted from the leaves of Westar and ZS5 plants inoculated with *Xcc* for 0, 5, and 8 days. The extracted metabolites were subsequently analyzed using LC-MS in combination with GC-MS methods. A total of 7604 metabolites belonging to 10 classes were detected (Table S7). PCA showed significant differences among groups, with biological replicates from the same group clustering closely together (Figure 4A), indicating the reproducibility and suitability of the metabolite data for further analyses. Differential accumulation analysis identified a total of 422 differentially accumulated metabolites (DAMs) with |log_2_FC| ≥ 1 and VIP ≥ 1 detected in Westar at 5 dpi, and 581 DAMs at 8 dpi (Figure 4B and Tables S8 and S9). In ZS5, a total of 318 DAMs were detected at 5 dpi, and 385 DAMs at 8 dpi (Figure 4B and Tables S10 and S11). Consistent with transcriptome data, a greater number of DAMs were observed in Westar compared to ZS5, with both varieties exhibiting an increase in the number of DAMs with prolonged infection time.

We conducted a KEGG functional analysis to investigate the metabolic alterations in rapeseed following *Xcc* infection. In Westar, 26 pathways were significantly enriched, while 23 pathways were significantly enriched in ZS5. Among them, 13 pathways were commonly enriched across both varieties, including ascorbate and aldarate metabolism, glyoxylate and dicarboxylate metabolism, tryptophan metabolism, and others (Table S12). The KEGG pathway analysis of DAMs revealed that both primary and secondary metabolites underwent substantial changes in response to *Xcc* infection in both *Xcc*-susceptible and *Xcc*-resistance plants (Figure 4C–F). Moreover, the DAMs and enriched pathways identified in Westar and ZS5 differed considerably, indicating their distinct responses to *Xcc* infection.

### 2.4. Identification of Metabolites That Mediate Xcc Resistance

**Figure 5:**
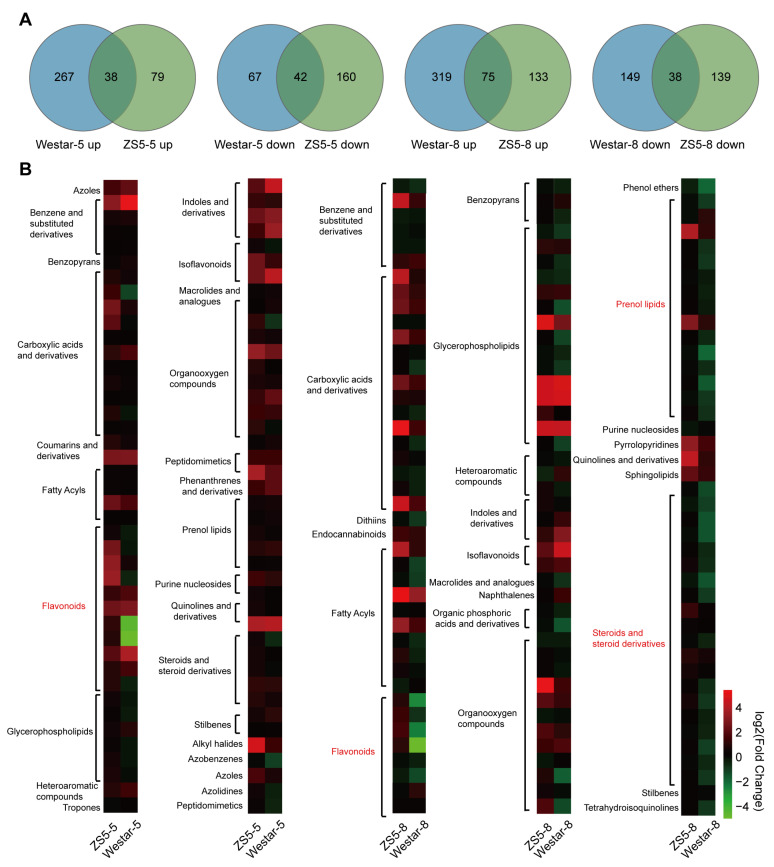
Unique DAMs in ZS5 varieties after *Xcc* infection. (**A**) Venn diagrams displaying the unique DAMs after *Xcc* infection for 5 days and 8 days. (**B**) Heat map showing the expression of unique DAMs in ZS5 after *Xcc* infection for 5 days and 8 days.

To further investigate the metabolic similarities and differences between Westar and ZS5 in response to *Xcc* infection, we performed a comparative analysis of their differential metabolites. Venn diagrams were utilized to identify the unique and common DAMs between Westar and ZS5. At 5 dpi, 343 unique DAMs were identified in Westar, including 276 up-regulated DAMs and 67 down-regulated DAMs. In contrast, ZS5 exhibited 239 unique DAMs, with 79 up-regulated and 160 down-regulated (Figure 5A). At 8 dpi, 468 unique DAMs including 319 up-regulated DAMs and 149 down-regulated DAMs were identified in Westar. Conversely, ZS5 showed 272 unique DAMs, with 133 up-regulated and 139 down-regulated (Figure 5A). These unique DAMs might play a critical role in resistance or susceptibility of the two varieties to *Xcc*.

To identify the potential metabolites involved in mediating resistance to *Xcc*, a heat map was generated to characterize the variations and patterns of unique DAMs identified in ZS5 (Figure 5B). The unique DAMs in ZS5 included various compounds such as carboxylic acids and derivatives, fatty acyls, flavonoids, indoles and derivatives, prenol lipids, organooxygen compounds, steroids, steroid derivatives, and others (Figure 5B). Notably, most DAMs belonged to prenol lipids, steroids, and steroid derivatives, and particularly flavonoids were up-regulated in ZS5 but down-regulated in Westar following *Xcc* infection (Figure 5B). For instance, some flavonoids, including cyanidin, malvidin 3-glucoside-pyruvate, and vitisidin A, were 6-, 10-, and 11-fold induced up-regulated at 5 dpi, and 5-, 5-, and 8-fold at 8 dpi in ZS5, whereas these compounds were down-regulated in Westar (Table S13). These findings indicated that flavonoid metabolites might play a crucial role in conferring resistance to *Xcc*.

### 2.5. Joint Analysis of Transcriptome and Metabolome

**Figure 6:**
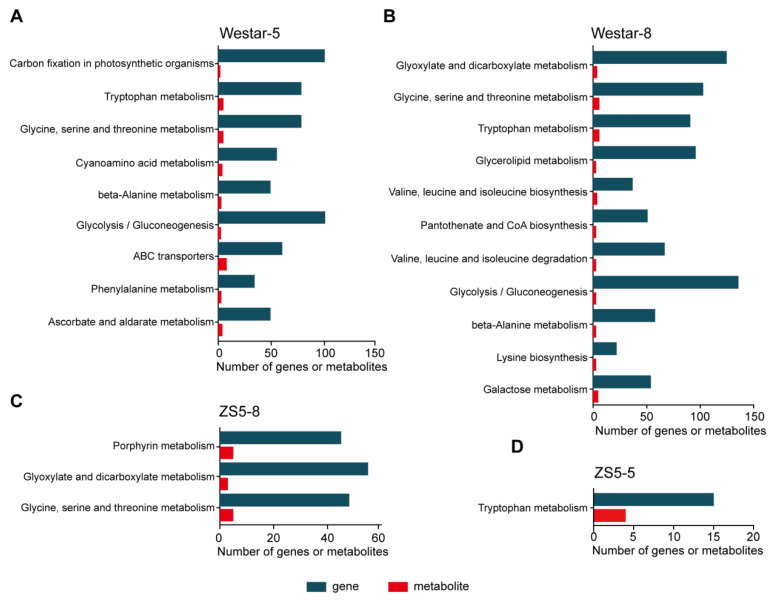
Transcriptomic and metabolomic joint analysis of Westar and ZS5 after *Xcc* infection. KEGG enrichment analysis of the DEGs and DAMs detected from Westar-5 (**A**), Westar-8 (**B**), ZS5-8 (**C**), and ZS5-5 (**D**).

To further understand the regulatory network of rapeseed in response to *Xcc* infection, we conducted a combined transcriptomic and metabolomic analysis and compared the enrichment of DEGs and DAMs in the Westar and ZS5 varieties. In Westar, DEGs and DAMs were significantly enriched in photosynthesis, tryptophan metabolism, glycine, serine and threonine metabolism, cyanoamino acid metabolism, beta-alanine metabolism, phenylalanine metabolism, ABC transporters, and so on (Figure 6A,B). In ZS5, the DEGs and DAMs were significantly enriched in tryptophan metabolism; porphyrin metabolism; glyoxylate and dicarboxylate metabolism; and glycine, serine, and threonine metabolism (Figure 6C,D). Importantly, the tryptophan metabolism pathway was significantly enriched both in Westar and ZS5 at 5 dpi, and also in Westar at 8 dpi (Figure 6).

### 2.6. Tryptophan-Derived Indole Glucosinolates Were Induced by Xcc Infection

**Figure 7:**
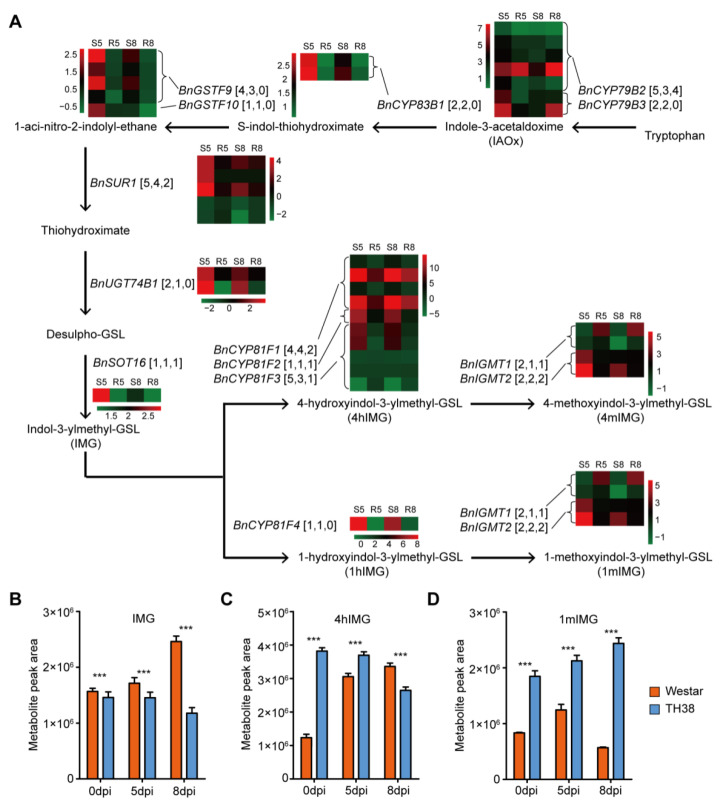
Indole glucosinolates in rapeseed are related to resistance to *Xcc*. (**A**) The expression of genes involved in the biosynthesis of indole glucosinolates in the leaves of the Xcc-susceptible variety (Westar) and Xcc-resistant variety (ZS5). The designations S5: Westar-5; S8: Westar-8; R5: ZS5-5; and R8: ZS5-8 are utilized for clarity. The numbers of gene copies and differentially expressed genes in Westar and ZS5 are listed in square brackets. Solid arrows represent pathways with identified substrates and enzymes, while dashed arrows denote products only. Abbreviations are provided in parentheses. (**B**–**D**) The levels of indole glucosinolates in rapeseed leaves before and after inoculation with *Xcc*. Data are means (three individual replicates) ± standard deviation (SD). Asterisks denote significant differences between Westar and ZS5. ***, *p* < 0.001 as determined by Student’s *t* test.

The integrated analyses indicated that tryptophan metabolism is crucial for the resistance of rapeseed to *Xcc*. Tryptophan (Trp) serves as a precursor for indole glucosinolates, which are synthesized through a series of enzymatic reactions leading to the production of 4-methoxyindol-3-ylmethyl glucosinolate (4mIMG) and 1-methoxyindol-3-ylmethyl glucosinolate (1mIMG) (Figure 7A). Previous studies have identified indol-3-ylmethylglucosinolate (IMG), 4-hydroxyindol-3-ylmethyl glucosinolate (4hIMG), 4mIMG, and 1mIMG as the four predominant types of indole glucosinolates [23]. In the DEGs obtained from the Westar and ZS5 varieties following *Xcc* infection, the expression of nearly all genes encoding catalytic enzymes involved in indole glucosinolates biosynthesis, as well as the transcription factors regulating this process, was significantly up-regulated at 5 dpi and/or 8 dpi in both Westar and ZS5 (Figure 7A and Figure S3). The expression patterns of these genes were validated by quantitative reverse transcription PCR (qRT-PCR) assay (Figure S4). Subsequently, the levels of indole glucosinolates in leaves were quantified using ultra performance liquid chromatography–tandem mass spectrometry (UPLC-MS/MS). The results revealed that IMG and 4hIMG were induced up-regulated in Westar but down-regulated in ZS5 during *Xcc* infection (Figure 7B,C). In contrast, 1mIMG was up-regulated in ZS5 during *Xcc* infection, while its levels decreased in Westar at 8 dpi. Moreover, the level of 1mIMG in ZS5 was approximately double that in Westar at both 0 dpi and 5 dpi, whereas at 8 dpi, it exhibited a more than fourfold increase compared to Westar (Figure 7D). Additionally, the level of 4hIMG, the precursor to 4mIMG, was more than threefold higher in ZS5 than in Westar at 0 dpi (Figure 7C). These results suggested that indole glucosinolates might play an important role in enhancing rapeseed resistance to *Xcc*, with variations in both the content and induction patterns between resistant and susceptible varieties.

### 2.7. Function of IAA in Mediating Rapeseed Resistance to Xcc

**Figure 8:**
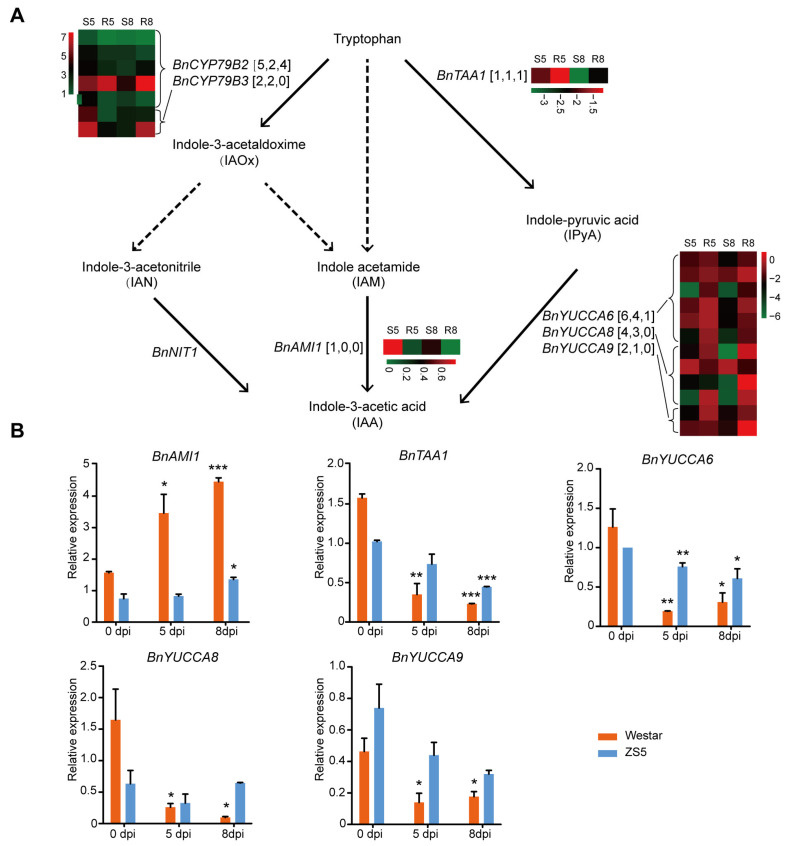
The expression of genes related to IAA biosynthesis within trp-dependent pathways in the leaves of the *Xcc*-susceptible variety (Westar) and *Xcc*-resistant variety (ZS5). (**A**) The expression data were obtained from transcriptomic analysis. Solid arrows denote pathways in which the enzymes, genes, or intermediates are known, while dashed arrows represent pathways that remain inadequately characterized. The numbers of gene copies and differentially expressed genes in Westar and ZS5 are listed in square brackets. Abbreviations are provided in parentheses. The designations S5: Westar-5; S8: Westar-8; R5: ZS5-5; and R8: ZS5-8 are utilized for clarity. (**B**) The relative expression data obtained from qRT-PCR. BnAction2 was used as the reference control. The data represent means (three biological repeats) ± SD. Asterisks denote significant differences revealed by Student’s *t* test (*, *p* < 0.05; **, *p* < 0.01; ***, *p* < 0.001).

In plants, the auxin indole-3-acetic acid (IAA) is primarily synthesized through a trp-dependent pathway, although an alternative trp-independent pathway also exists [25]. Within the trp-dependent pathway, tryptophan aminotransferase (TAA) converts Trp to indole-3-pyruvic acid (IPyA), which is subsequently transformed into IAA by the YUCCA (YUC) favin monoxygenase. Additionally, the CYP79B2/3 enzymes convert Trp to indole-3-acetaldoxime (IAOx), which undergoes further enzymatic catalysis to produce intermediate compounds such as indole acetamide (IAM) or indole-3-acetonitrile (IAN). These intermediates are then converted into IAA via the catalytic activity of amidase1 (AMI1) and nitrilase1 (NIT1), respectively (Figure 8A). The IPyA pathway is a major and essential route for IAA biosynthesis in plants [26]. In the DEGs identified from Westar and ZS5 plants following *Xcc* infection, the expression of genes in the IPyA pathway were all down-regulated (Figure 8). The qRT-PCR results revealed a significantly greater down-regulation expression of *BnTAA1*, *BnYUCCA6*, *BnYUCCA8*, and *BnYUCCA9* in Westar compared to ZS5 (Figure 8B). In addition, the expression of *BnCYP79B2*, *BnCYP79B3*, and *BnAMI1* were up-regulated in both Westar and ZS5 (Figure 8B and Figure S4).

**Figure 9:**
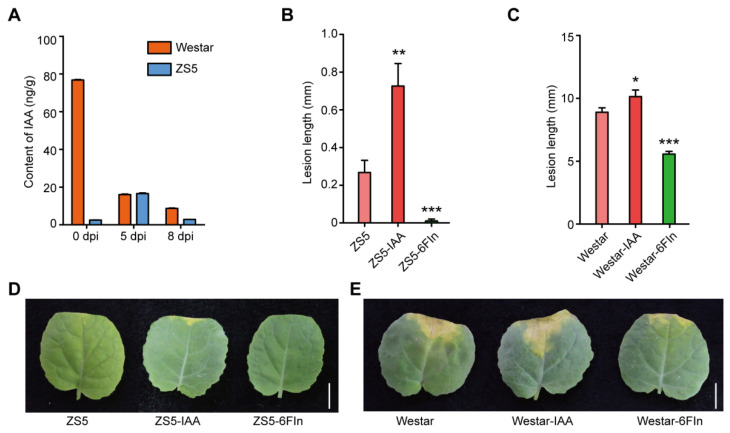
Indole 3-acetic acid (IAA) negatively regulated rapeseed resistance to *Xcc*. (**A**) The changes of IAA content in Westar and ZS5 during *Xcc* infection. Data are means (three individual replicates) ± SD. (**B**,**C**) The phenotype of resistance to *Xcc* after treatment with ddH_2_O, 200 µM IAA, and 200 µM 6-fluoroindole applied to the leaves of ZS5 (**B**) and Westar (**C**). Statistical significance is indicated as follows: *, *p* < 0.05; **, *p* < 0.01; ***, *p* < 0.001 by Student’s *t* test. (**D**,**E**) The representative images corresponding to (**B**,**C**). Scale bar = 1 cm.

To determine the role of IAA in the resistance of rapeseed to *Xcc*, we measured the content of IAA in Westar and ZS5 leaves during *Xcc* infection. In Westar, the IAA content decreased dramatically from 76.80 ng/g to 16.02 ng/g at 5 dpi and further dropped to 8.69 ng/g at 8 dpi following *Xcc* infection. In contrast, in ZS5 plants, the IAA content initially increased from 2.54 ng/g to 16.54 ng/g at 5 dpi and then declined to 2.84 ng/g at 8 dpi (Figure 9A). Notably, it was observed that ZS5 had lower levels of IAA compared to Westar (Figure 9A). These findings indicated that *Xcc* infection inhibited IAA biosynthesis in Westar but induced an initial increase followed by a decrease in ZS5. Furthermore, we conducted experiments involving the exogenous application of both IAA and the IAA synthesis inhibitor 6-fluoroindole on seedlings of Westar and ZS5. At 8 dpi, when minor disease symptoms occurred around the inoculation site on ZS5 leaves, the lesion length was approximately 1 mm for ZS5 plants treated with exogenous IAA (Figure 9B,D). However, the application of the IAA synthesis inhibitor on ZS5 effectively prevented *Xcc* infection in the leaves (Figure 9B,D). Similarly, the resistance of Westar to *Xcc* was enhanced following treatment with the IAA synthesis inhibitor, whereas Westar displayed increased susceptibility to *Xcc* when treated with IAA (Figure 9C,E). These findings revealed that IAA negatively regulates resistance to *Xcc* in both ZS5 and Westar.

### 2.8. qRT-PCR Verification of Gene Expression

To validate the credibility of the expression levels obtained from the RNA-seq data, qRT-PCR was performed on the DEGs involved in tryptophan-derived indole glucosinolates and trp-dependent IAA synthesis pathway (Table S14). The qRT-PCR results for all 18 genes exhibited a high degree of consistency with the transcriptome data (Figure S4 and Figure 8B). The average correlation coefficient between the qRT-PCR and the transcriptome data was calculated to be 0.80 (Table S15), thereby confirming the reliability of our RNA-seq findings.

## 3. Discussion

Black rot, a bacterial disease caused by *Xanthomonas campestris* pv. *campestris* (*Xcc*), is one of the most destructive diseases affecting *Brassica* crops worldwide, resulting in substantial losses in both yield and quality [3]. Breeding resistant varieties is regarded as the most effective strategy for managing this disease, which requires a comprehensive understanding of the interactions between rapeseed and *Xcc*, as well as the identification of resistance-related genes. This study integrated transcriptome and metabolome analyses to uncover molecular mechanisms underlying resistance in two rapeseed varieties with contrasting resistance levels to *Xcc*, exploring the candidate genes and metabolites that contribute to resistance in rapeseed.

In analyses of transcriptome and metabolome, the numbers of DEGs and DAMs in both resistant and susceptible varieties of rapeseed were increasing over the course of infection, suggesting a progressive enhancement of defense responses. The DEGs identified in both resistant and susceptible rapeseed varieties were predominantly enriched in phenylpropanoid biosynthesis, cutin, suberine and wax biosynthesis, tryptophan metabolism, and phenylalanine metabolism. However, there were obvious differences in the responses of resistant and susceptible rapeseed varieties following *Xcc* infection. Susceptible rapeseed exhibited more pronounced transcriptomic and metabolomic changes, as evidenced by the identification of a greater number of DAMs and DEGs than the resistant variety. Additionally, compared with susceptible rapeseed, the DEGs in resistant rapeseed were also enriched in defense response and flavonoid biosynthesis in the early stage of infection. Furthermore, most DAMs belonging to flavonoids were up-regulated in resistant rapeseed but down-regulated in susceptible rapeseed following *Xcc* infection. Flavonoids are widely distributed secondary metabolites, preventing plants from pathogens infection and insects feeding [27,28]. Numerous studies have revealed that antibacterial mechanisms of flavonoids are included mainly by the inhibition of synthesis of nucleic acid; by the inhibition of cytoplasmic membrane function by influencing biofilm formation, porins, and permeability; and by the interaction with some crucial enzymes [29,30,31]. Therefore, flavonoids may contribute to the enhanced resistance of rapeseed to *Xcc* by inhibiting bacterial proliferation.

Photosynthetic metabolism plays a significant role during plant–pathogen interactions. Suppression of photosynthesis is an effective plant defense mechanism against biotrophic pathogens [32,33]. Previous studies indicated that the majority of photosynthesis-related proteins and genes were down-regulated in resistant *B. oleracea* plants but up-regulated in susceptible plants [15,34]. In this study, transcriptome analysis revealed that photosynthesis was significantly down-regulated in both the early and late stages of susceptible rapeseed, as well as in the late stage of resistant rapeseed infected by *Xcc.* The decline in photosynthetic activity was attributed not only to cell death but also to modifications in the host carbon utilization [35]. The observed down-regulation of DEGs involved in photosynthesis in susceptible rapeseed plants, along with the late stage of resistant rapeseed plants, suggested that the rapeseed plants may try to control the energy supply of *Xcc* by reducing the photosynthetic metabolism, thereby inhibiting its growth.

Integrated transcriptome and metabolome analyses revealed that the tryptophan metabolism pathway is implicated in the resistance of rapeseed to *Xcc*. Tryptophan serves as a precursor of indole glucosinolate, which is an important type of glucosynolates in *Brassica* species [36]. Glucosinolates are recognized as crucial secondary metabolites implicated in plant defense against pathogens and herbivorous insects [37]. When a pathogen causes tissue damage, the enzyme myrosinase hydrolyzes glucosinolates into various products that exhibit antimicrobial activity against bacterial and fungal plant pathogens in vitro [38,39,40]. The overexpression of the indolic glucosinolates pathway gene *UGT74B* in *B. napus* enhanced the overall production of indolic glucosinolates, thereby increasing resistance to *Sclerotinia sclerotiorum* and *Botrytis cinerea* [41]. Our findings revealed that the expression levels of nearly all genes in the indolic glucosinolate pathway, as well as the content of indolic glucosinolates, were significantly elevated in both susceptible and resistant rapeseed varieties following *Xcc* infection. Moreover, resistant plants exhibited higher accumulation of these compounds, suggesting their critical role in enhancing resistance to *Xcc*. We propose that the increased levels of indolic glucosinolates in the *Xcc*-resistant rapeseed variety contribute to its enhanced resistance relative to the susceptible variety.

Tryptophan serves as a precursor not only for indole glucosinolides but also for the synthesis of IAA, which is the predominant form of auxin in plants and important for plant growth and development [42]. The exogenous application of IAA has been shown to increase the lesion areas in resistant rice lines infected with *Xanthomonas oryzae* pv. *oryzae* [20]. Additionally, external IAA treatment aggravates disease progress caused by *Xanthomonas oryzae* pv. *oryzicola* and *Magnaporthe oryzae* in rice [43]. The auxin transport inhibitor can attenuate the development of canker in sweet orange infected by *Xanthomonas axonopodis* pv. *citri* [44]. In experiments with *B. napus* seedlings, exogenous IAA promoted the development of clubroot disease, whereas treatment with the IAA polar transport inhibitor attenuated the disease [45]. Overall, exogenous application of IAA can promote disease development during plant–pathogen interactions. In this study, transcriptome analysis and qRT-PCR revealed that the genes in the IPyA pathway, which is dependent on tryptophan for IAA synthesis, were down-regulated in both resistant and susceptible rapeseed varieties. Furthermore, the IAA content decreased dramatically in the susceptible rapeseed variety following *Xcc* infection. In contrast, the resistant rapeseed variety exhibited an initial increase in IAA level, followed by a decline at a later stage. Overall, the content of IAA was lower in the resistant rapeseed than susceptible rapeseed. The exogenous application of IAA resulted in increased lesion areas in both resistant and susceptible rapeseed varieties inoculated with *Xcc*. Moreover, the application of an IAA synthesis inhibitor can attenuate the development of black rot in both resistant and susceptible rapeseed varieties. The plant cell wall serves as a natural protective barrier against pathogens; however, IAA can weaken this barrier by increasing cell wall extensibility, thereby facilitating pathogen invasion and increasing plant susceptibility [20,46,47]. Consequently, it can be inferred that IAA negatively regulates resistance to *Xcc* in rapeseed by weakening the plant cell wall, creating an opportunity for *Xcc* attack.

Plants have developed intricate defense mechanisms to combat a variety of pathogens over the long period of their evolution. Tryptophan, a crucial amino acid in protein synthesis, is synthesized in plants and subsequently contributes to the formation of growth regulatory substances and various secondary metabolites. These compounds are essential for plant growth, development, and the defense against biological stress [48]. In this study, we found that two tryptophan-derived metabolites play important roles in black rot resistance of rapeseed. Specifically, indolic glucosinolates were found to positively regulate resistance, while IAA negatively regulates resistance. Overall, our findings provide novel insights into plant–pathogen interactions and may help to improve rapeseed resistance against the pathogen.

## 4. Materials and Methods

### 4.1. Plants and Growth Conditions

Two *B. napus* varieties were used in this study. The Westar variety was a widely cultivated spring type and susceptible to *Xcc*. In contrast, Zhongshuang 5 (ZS5), a semi-winter variety, is resistant to *Xcc*. The plant materials were grown in a greenhouse under controlled conditions, specifically at temperatures of 22/18 °C (day/night), a photoperiod of 16/8 h, and relative humidity levels exceeding 60–70%.

### 4.2. Xcc Resistance Evaluation

*Xcc* race 3 were grown on LB medium plates, which consisted of 10 g/L tryptone, 5 g/L yeast extract, 10 g/L NaCl, and 15 g/L agar (pH 7.0) and maintained at 28 °C for 48 h. Subsequently, fresh bacterial cultures were dissolved in sterilized double-distilled water (ddH_2_O) and adjusted to an OD_600_ value of 0.3. To enhance bacterial adhesion, 2 mL/L Tween 20 was added before infection. Disease assays were conducted using plants at the four-leaf stage, wherein three leaves per plant were inoculated by clipping leaf tips (approximately 2 cm) with scissors that had been dipped in the bacterial suspension. Control leaves were treated similarly, but with scissors dipped in ddH_2_O. After inoculation, greenhouse conditions were optimized to facilitate *Xcc* infection, maintaining a temperature of 28/24 °C (day/night), a photoperiod of 14/10 h, and relative humidity exceeding 90%. At 8 days post-inoculation (dpi), the depths of the V-shaped lesions were measured, and the mean value, excluding the extreme lengths of the lesions, was used as the indicator of disease resistance phenotype.

### 4.3. RNA-Seq and Data Analysis

The leaves of Westar and ZS5 at the four-leaf stage were used for RNA sequencing. Each seedling was inoculated with *Xcc* for durations of 5 and 8 days, serving as the experimental groups, and seedlings that were inoculated with ddH_2_O served as control groups. At 5 and 8 dpi, the margin of approximately 5 mm of seemingly unaffected tissue was sampled from each inoculated true leaf using surgical scissors. All samples were subsequently frozen in liquid nitrogen and stored at −80 °C until further use. Each sample comprised three biological replicates with four plants per replicate. RNA was extracted from the samples using the FastPure Plant Total RNA Isolation Kit (Vazyme, Nanjing, China), and the resulting RNA was assessed for quality before being employed for transcriptome sequencing on the Illumina platform. The clean reads obtained from sequencing were aligned to the *Brassica napus* reference genome Westar [49] (https://bnaomics.ocri-genomics.net/organism/10327, accessed on 30 December 2024) using Hisat2 version 2.0.5 [50]. To quantify the expression of assembled transcripts, the transcripts were analyzed using StringTie version 2.2.3 [51] as fragments per kilobase of exon model per million mapped reads (FPKM). Differential expression analysis was performed using DESeq2 version 1.20.0 [52], and genes with expression differences meeting the thresholds of |log_2_(Fold Change)| > 1 and padj < 0.05 were designated as differentially expressed genes (DEGs).

### 4.4. Function Analysis of DEGs

DEGs obtained through the above methods were compared against the whole genomic background utilizing a hypergeometric test for Gene Ontology (GO) and Kyoto Encyclopedia of Genes and Genomes (KEGG) functional analysis. The hypergeometric test was corrected by the Benjamini and Hochberg false discovery rate, with a significance threshold set at 0.05.

### 4.5. Metabolomics and Data Analysis

The samples used in the metabolomic analysis were the leaves of Westar and ZS5 at the four-leaf stage. The sampling method employed was consistent with that used for transcriptome sequencing. The leaf samples were subjected to untargeted metabolomic analyses, specifically gas chromatography–mass spectrometry (GC-MS) and liquid chromatography–mass spectrometry (LC-MS), conducted by Shanghai Luming Biological Technology Co., Ltd., Shanghai, China. Each sample contained three biological replicates with four plants per replicate. A comprehensive description of the experimental methods and metabolomic analyses is provided in reference [53]. Differentially accumulated metabolites (DAMs) were identified based on variable importance in projection (VIP) > 1 and *p* value < 0.05.

### 4.6. RNA Isolation and qRT-PCR Analysis

The samples used for qRT-PCR analysis were the leaves from Westar and ZS5 at the four-leaf stage. Total RNA was extracted from the powdered tissue using TRIzol reagent. Subsequently, reverse transcription of the RNA into cDNA was performed employing the PrimeScrip RT Reagent Kit (Takara, San Jose, CA, USA, RR047Q). Gene expression detection was carried out using SYBR Green PCR Master Mix on the CFX96 Real-Time System (Bio-Rad, Hercules, CA, USA). BnActin2 was selected as the internal reference gene. The mean and standard error were calculated from three biological replicates, with each replicate subjected to triplicate technical repeats during PCR. The primer sequences used in this study were detailed in Table S14.

### 4.7. Indole Glucosinolate Content Measurements

To detect the content of indole glucosinolate, leaves of the Westar and ZS5 varieties at the four-leaf stage were collected. Then, the samples were subsequently processed into powder using vacuum freeze-drying technology. Following this, 50 mg of the powdered sample was added 1200 μL of pre-cooled 70% methanolic aqueous internal standard extract. The sample was filtered and stored in an injection vial for further analysis. The extracted metabolites were detected using ultra performance liquid chromatography–tandem mass spectrometry (UPLC-MS/MS) at Wuhan Maiwei Metabolic Biotechnology Co., Ltd., Wuhan, China.

### 4.8. IAA Content Measurements

The IAA contents in the leaves of Westar and ZS5 at the four-leaf stage were quantified. Initially, the leaves were ground into powder in liquid nitrogen, and a 0.2 g sample of this powder was used for IAA extraction by acetonitrile solution. Subsequently, 50 mg of C18 sorbent was added to the acetonitrile solution that dissolved IAA, followed by thorough mixing and centrifugation. The supernatant was collected and evaporated to dryness under a stream of nitrogen gas. Finally, 200 mL methanol was added to the sediment for complete dissolution, and the solution was filtered using 0.22 μM organic phase filter membrane. The IAA content was then detected using high-performance liquid chromatography–tandem mass spectrometry (HPLC-MS/MS) at Wuhan Pronets Testing Technology Co., Ltd., Wuhan, China.

### 4.9. Exogenous IAA and IAA Inhibitor Treatment

To investigate the effect of indole 3-acetic acid (IAA) on *B. napus* defenses to *Xcc*, a total of 32 Westar or ZS5 seedlings at the four-leaf stage were sprayed with IAA (200 µM), with water serving as the control. Subsequently, three leaves from each plant were inoculated with *Xcc*. IAA or water was reapplied at intervals of 2, 4, and 6 dpi. Furthermore, to evaluate the effects of the auxin synthesis inhibitor 6-fluoroindole on the defensive responses of *B. napus*, both Westar and ZS5 seedlings at the four-leaf stage were sprayed with 6-fluoroindole (200 µM) or water at intervals of 2, 4, and 6 dpi. At 8 dpi, the depths of V-shaped lesions were measured, and the mean value, excluding the extreme lengths of the lesions, was used as the indicator of disease resistance phenotype.

## 5. Conclusions

**Figure 10:**
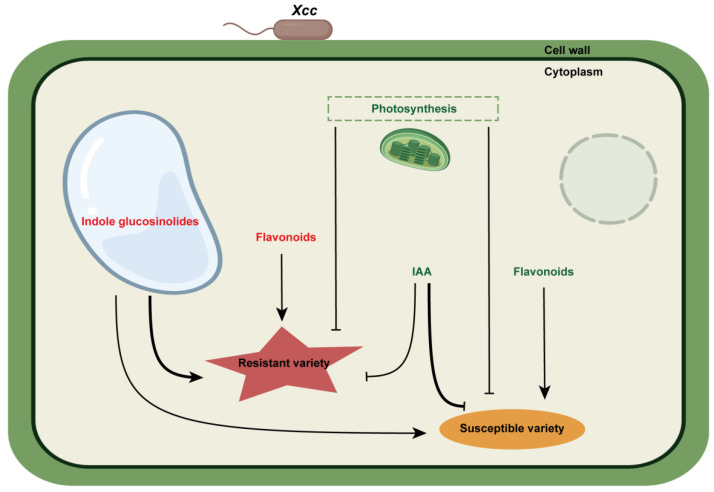
Schematic view of a working model of rapeseed’s defense responses to X. campestris infection from the integrated transcriptome and metabolome analysis. The names in red and green indicate metabolites or biological processes that were up- and down-regulated, respectively. The arrows represent positive regulation and the T-shaped arrows represent negative regulation. The thick line represents a higher metabolite content.

This study investigated the transcriptomic and metabolic changes in the leaves of susceptible rapeseed variety Westar and resistant rapeseed variety ZS5 upon *Xcc* infection. We proposed a working model of the rapeseed–*Xcc* interactions (Figure 10). Photosynthesis was down-regulated in the susceptible rapeseed post-infection, while this down-regulation occurred later in the resistant rapeseed. Flavonoids were up-regulated in the resistant rapeseed but down-regulated in the susceptible rapeseed following *Xcc* infection. The tryptophan metabolism pathway was significantly enriched in both varieties. Indolic glucosinolates were found to positively regulate rapeseed resistance, and the resistant rapeseed exhibited higher accumulation of indolic glucosinolates. IAA negatively regulated rapeseed resistance, and the content of IAA was lower in the resistant rapeseed than the susceptible rapeseed. In summary, rapeseed plants may try to increase flavonoids and indolic glucosinolate biosynthesis, reduce IAA biosynthesis, and control the energy supply of *Xcc* by reducing the photosynthetic metabolism in order to inhibit *Xcc* growth (Figure 10). Our findings provide valuable molecular insights into the interactions between rapeseed and *Xcc*, facilitating the breeding of black-rot-resistant *Brassica* crops.

## Data Availability

The raw sequence data reported in this study are deposited in the Genome Sequence Archive in National Genomics Data Center (GSA: CRA021687) that are publicly accessible at https://ngdc.cncb.ac.cn/gsa (accessed on 30 December 2024).

## Supplementary Materials

The supporting information can be downloaded at https://www.mdpi.com/article/10.3390/ijms26010367/s1.
