# Supplementary material for: Integrated Transcriptome and Metabolome Analysis Reveals the Resistance Mechanisms of Brassica napus Against Xanthomonas campestris
Source: Int J Mol Sci. 2025 Jan 3;26(1):367. doi: 10.3390/ijms26010367 (PMC11721368; doi:10.3390/ijms26010367)
Supplement: Supplementary file 1 [file ijms-26-00367-s001.zip › ijms-3385302-supplementary/Supplementary files/Supplementary Figures.pdf]

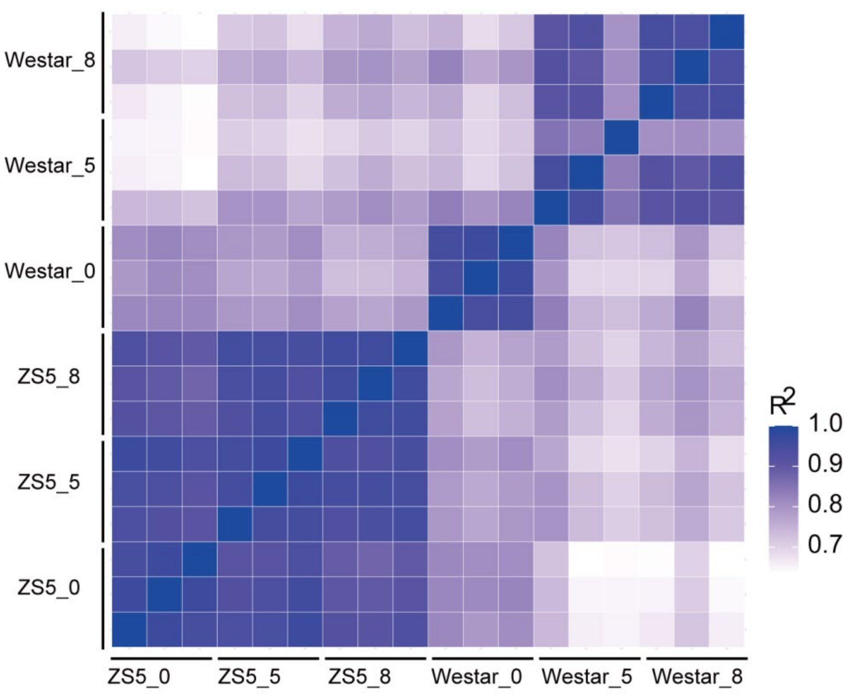

Figure S1. Correlation analysis of FPKM values between Westar and ZS5.

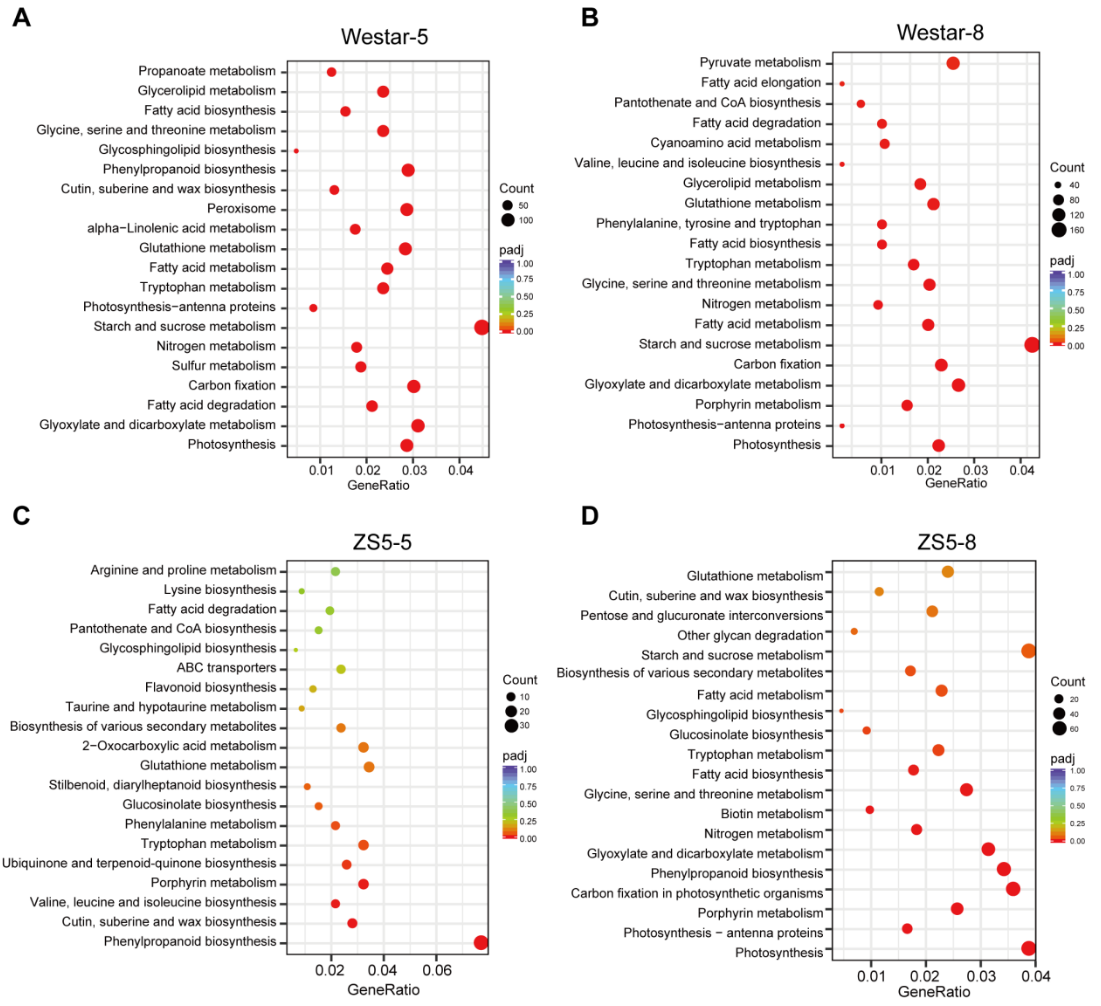

Figure S2. KEGG functional analysis of DEGs from the two varieties after *Xcc* infection compared with uninfected control. KEGG pathways enriched in Westar (A-B) and ZS5 (C-D), respectively.

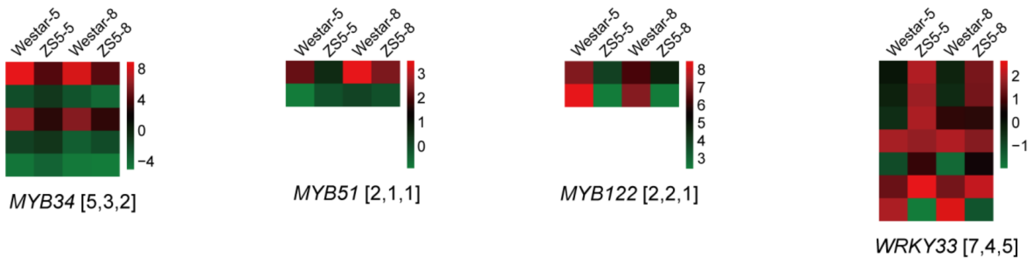

Figure S3. The expression of transcription factors that regulate indole glucosinolates biosynthesis in the leaves of *Xcc*-susceptible line (Westar) and *Xcc*-resistant line (ZS5). The copy numbers of the genes and the differentially expressed genes in Westar and ZS5 are listed in square brackets.

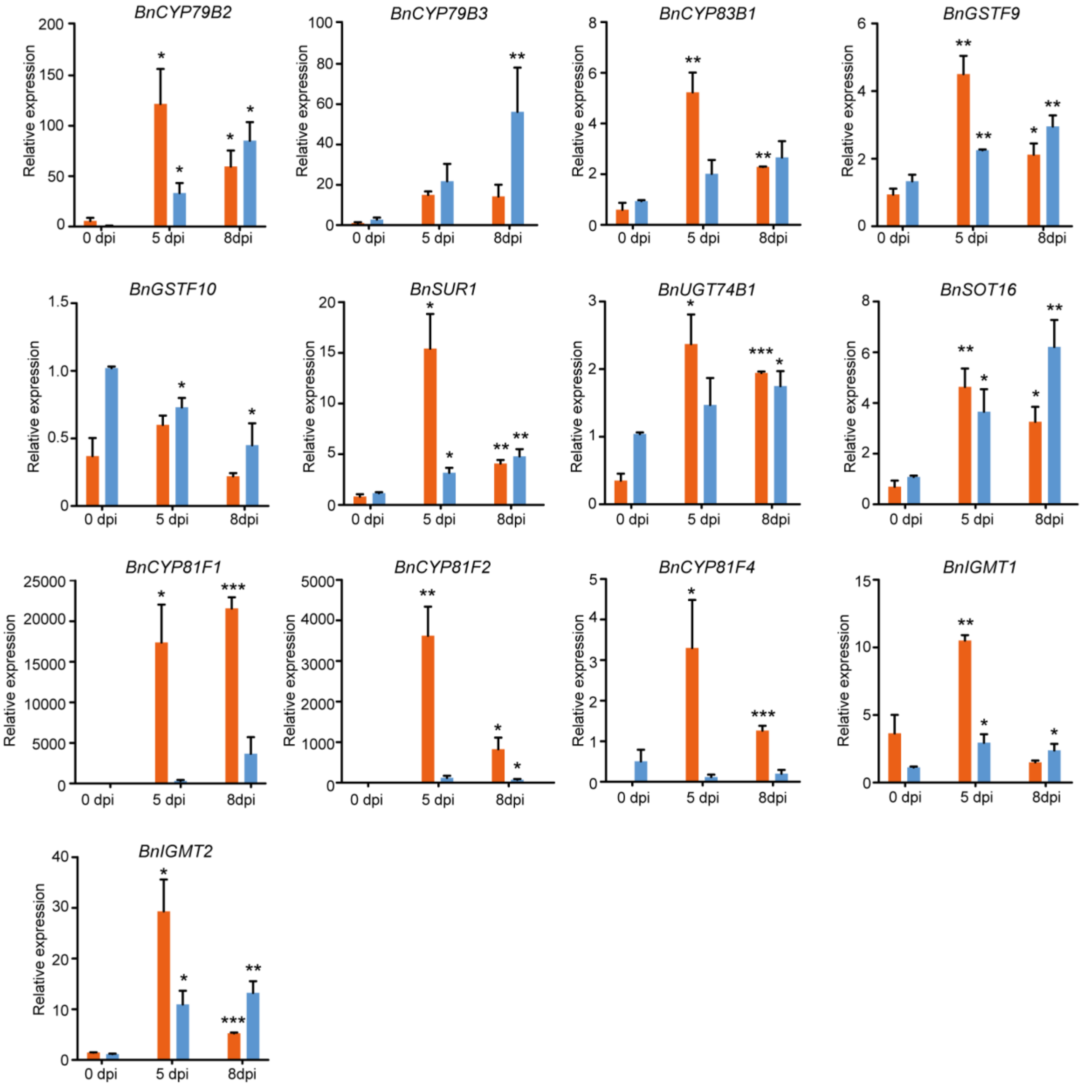

Figure S4. The relative expression of genes related to indole glucosinolates biosynthesis in the leaves of *Xcc*-susceptible line (Westar) and *Xcc*-resistant line (ZS5). BnAction2 was used as a reference control. Data represent means (three biological repeats) ± SD. Asterisks indicate significant differences revealed by Student's t test (*, $p<0.05$; **, $p<0.01$; ***, $p<0.001$).
